# Inhibited oligodendrogenesis impairs attention and motivation in adult mice regardless of repeated mild traumatic brain injury

**DOI:** 10.3389/fnbeh.2026.1862945

**Published:** 2026-08-31

**Authors:** Lisa M. Gazdzinski, Jordan Mak, Miranda Mellerup, Paul John Fletcher, Anne L. Wheeler

**Affiliations:** 1 Neurosciences and Mental Health, The Hospital for Sick Children, Toronto, ON, Canada; 2 Department of Physiology, University of Toronto, Toronto, ON, Canada; 3 Departments of Psychiatry and Psychology, University of Toronto, Toronto, ON, Canada; 4 Centre for Addiction and Mental Health, Toronto, ON, Canada

**Keywords:** attention, motivation, myelin, oligodendrogenesis, traumatic brain injury

## Abstract

**Introduction:**

The brain’s white matter is responsible for communication across the brain and because attention relies on coordinated communication among distributed brain networks, white matter dysfunction may contribute to attentional impairment. Attention problems are among the most common long-lasting cognitive symptoms of mild traumatic brain injury (mTBI) and as attention is fundamental to many aspects of cognition, the effects of attentional impairment can be broad. mTBI-induced effects on oligodendrocytes and myelin contribute to cognitive deficits following injury and myelin plasticity is a potential mechanism for functional recovery. The aims of this work were to investigate the effects of mTBI and inhibition of oligodendrogenesis on attention in mice, and evaluate the contribution of newly generated oligodendrocytes to behavioral recovery following injury.

**Methods:**

This study used the Myrf conditional knockout mouse model, in which the Myrf gene, required for oligodendrocyte precursor cell (OPC) differentiation into mature myelinating oligodendrocytes, is deleted from OPCs following tamoxifen injection, thereby halting oligodendrogenesis. Mice were trained on the 5-Choice Serial Reaction Time (5-CSRT) task before receiving tamoxifen followed by three mTBI or sham procedures. Attention was probed on the 5-CSRT task with decreasing stimulus duration at four timepoints following injury out to 12 weeks.

**Results:**

While no mTBI-induced attentional impairment was observed, OPC-MyrfKO mice showed lower accuracy (β = −4.26%, p_*Bonf*_ = 0.003) across injury groups, timepoints and stimulus durations, and fewer premature trials at the later timepoints (genotype-by-timepoint interaction p_*Bonf*_ = 0.03; β = −4.8%, *p* = 0.017 at 8 weeks and β = −5.19%, *p* = 0.027 at 12 weeks), suggesting that active oligodendrogenesis is required for sustained attention and task engagement.

**Conclusion:**

These results suggest that myelin plasticity in adulthood may contribute to attention and complex task performance. The mouse model of mTBI used did not impact attention as measured by the 5-CSRT task, thus further research is required to elucidate the role of oligodendrogenesis in post-mTBI cognitive recovery.

## 1 Introduction

White matter, comprised of bundles of axons, forms the structural connectivity that enables communication across the brain. Complex cognitive functions, such as learning, attention, executive function, memory, and language, rely on the coordination of distributed neural networks, which depend on the structural integrity and plasticity of the associated white matter (Filley and Fields, 2016). Multiple large-scale functional brain networks have been associated with various aspects of attention, and altered connectivity within these networks has been associated with a variety of pathology conditions, including brain injury (Han et al., 2016; Posner et al., 2016; Cook et al., 2025).

Mild traumatic brain injury (mTBI), also often called concussion, is a condition in which white matter damage is likely to contribute to cognitive symptoms, which may include attentional impairment. mTBI is very common, with an estimated 1 in 4 people being affected in their lifetime (Daugherty et al., 2020). Repeated injuries are common, particularly among contact sport athletes (Glaser et al., 2024; Yates et al., 2025) and in people who experience intimate partner violence (Smirl et al., 2019). While most recover fully within a few days to a couple of weeks, a significant proportion (∼30%) of people experience prolonged symptoms that can last months or years after the injury (Cancelliere et al., 2023). A recent systematic review listed impaired attention among the most common cognitive deficits following concussion from the acute through the chronic period (Collins et al., 2023). Attention deficits have also been reported in pre-clinical TBI models. Impaired performance on the 5-choice serial reaction time (5-CSRT) task has been observed in juvenile and adult rats following repeated mTBI (Mychasiuk et al., 2015; Xu et al., 2021; Vonder Haar et al., 2022), and a transient post-mTBI deficit has been reported in mice in the object-based attention test (Zhang et al., 2021) and in the Y-maze spontaneous alternation task (Gazdzinski et al., 2020), which is considered a test of working memory, but also requires attentional processes for performance. As attention is fundamental to many other aspects of cognition, attentional impairment can impact many domains of daily functioning for people after mTBI, thus therapeutic targets are needed.

White matter is particularly vulnerable to damage from TBI (Armstrong et al., 2016). Axons may be directly damaged by stretching and shearing forces imparted on them by the acceleration and deceleration of the head during the injuring event itself (Johnson et al., 2013), but if the injury is mild, it does not necessarily lead to axonal loss and cell death. Instead, mild trauma can result in damaged axons dispersed among intact ones within major white matter tracts (Povlishock, 1993; Sullivan et al., 2013). Many large caliber axons are surrounded by myelin sheaths. Myelin is formed from multiple layers of extended cell membrane from oligodendrocytes, with a single oligodendrocyte contributing myelin to several axons. In the damaged white matter environment that results from mTBI, oxidative stress, glutamate excitotoxicity, and inflammation can lead to loss of oligodendrocytes, demyelination of intact axons, and changes to myelin structure (Lotocki et al., 2011; Shi et al., 2016). Given myelin’s role in supporting and tuning neural circuit function, myelin damage may be a significant factor underlying the cognitive impairments following mTBI (Mierzwa et al., 2015). Fortunately, evidence for myelin plasticity and repair after mTBI has been found in both human imaging studies, which have shown evolution and in some cases normalization of myelin water fraction and diffusion MRI measures over time (Weber et al., 2018; Lindsey et al., 2023), and in animal models of mTBI, in which increased oligodendrocyte precursor cell (OPC) proliferation and evidence of remyelination have been reported (Sullivan et al., 2013; Mierzwa et al., 2015; Nonaka et al., 2021). Thus, myelin plasticity is a potential mechanism for functional recovery from mTBI and understanding its role is important for assessing it as a therapeutic target.

The objectives of the current study were to determine how repeated mTBI and inhibition of oligodendrogenesis influence attention, and to test whether adult-born oligodendrocytes contribute to behavioral recovery following injury. The study was conducted in Myrf conditional knockout (Myrf-cKO) mice, in which the myelin regulatory factor (Myrf) gene, a transcription factor required for OPC differentiation into myelin producing oligodendrocytes, is deleted from OPCs in a time-controlled manner via tamoxifen administration (McKenzie et al., 2014; Duncan et al., 2018; Pan et al., 2020; Steadman et al., 2020; He et al., 2024; Xin et al., 2024; Gazdzinski et al., 2025). Mice were trained on the 5-CSRT task, a pre-clinical adaptation of the continuous performance test commonly used to assess attention in humans (Mar et al., 2013; Fizet et al., 2016). After training, oligodendrogenesis was halted in Cre+ Myrf-cKO mice (OPC-MyrfKO) before the mice underwent repeated mTBI or sham procedures. Attention was assessed by decreasing the stimulus duration on the 5-CSRT task at four timepoints: 1, 4, 8, and 12 weeks post-mTBI. While attentional impairment was not observed following mTBI in this study, mice with disrupted oligodendrogenesis had lower accuracy, omitted more trials, and were slower to collect the reward across injury groups, stimulus durations and timepoints compared to Cre− controls (OPC-MyrfKO-CTRL), suggesting that active myelination is required for sustained attention and engagement in the task.

## 2 Materials and methods

### 2.1 Mice

**FIGURE 1:**
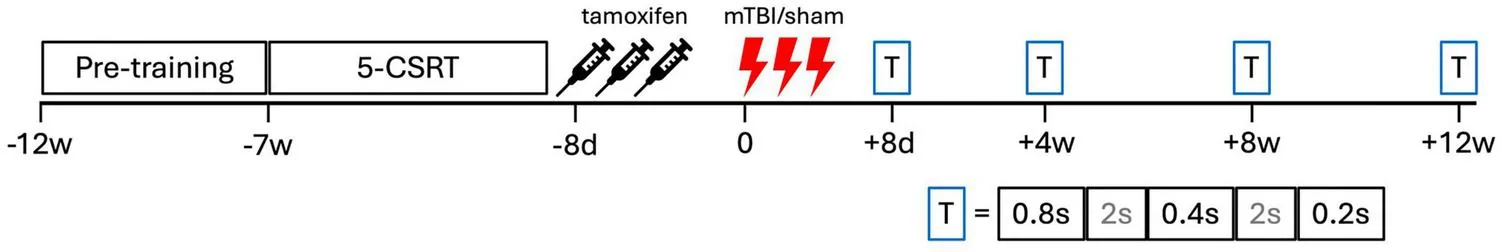
Study timeline. Mice underwent pre-training followed by 5-CSRT training starting about 12 weeks before mTBI. Tamoxifen was given 8 days before the first mTBI procedure to halt oligodendrogenesis in Cre+ Myrf-cKO mice (OPC-MyrfKO). Testing (T in blue box) was performed at 8 days, 4 weeks, 8 weeks and 12 weeks post-mTBI. At each testing timepoint, the probe trial stimulus duration decreased from 0.8 s to 0.4 s to 0.2 s with rebaselining sessions with 2-s stimulus duration between probe trials.

All animal procedures were approved by The Centre for Phenogenomics animal care committee. Myrf-cKO mice were generated by first crossing Myrf^*flox*/*flox*^ mice (The Jackson Laboratory #01067, in-house colony) with NG2-CreER*™* mice (The Jackson Laboratory #8538, in-house colony) to generate Myrf*^*flox*/+^*:NG2-CreER*™* mice, which were backcrossed with Myrf*^*flox*/*flox*^* mice to yield mice that were homozygous for the floxed Myrf allele (Myrf*^*flox*/*flox*^*) and either hemizygous (Cre+) or non-carrier (Cre−) for the NG2-CreER*™* gene. Approximately equal numbers of male and female mice of each Cre genotype were included in each injury group (*N* = 76; 18–20/genotype/injury group; detailed group sizes are shown in Table 1), and mice were housed in sex-separated cages in groups of 2–5. Pre-training began when mice were 8–11 weeks old. Training and testing were performed during the latter half of the light phase of the 12-h light/dark cycle (ZT6-ZT12). A study timeline is shown in Figure 1.

**TABLE 1:** Detailed group sizes.

Injury group	OPC-MyrfKO-CTRL (Cre−)		OPC-MyrfKO (Cre+)	
	Males	Females	Males	Females
Sham	8	10	10	9
mTBI	8	11	11	9

### 2.2 Tamoxifen administration

Tamoxifen (T5648 Sigma-Aldrich, 180 mg/kg dissolved at 30 mg/ml in 10% ethanol in sunflower seed oil (Sigma-Aldrich, Canada)), as used previously (Akers et al., 2014; Steadman et al., 2020; Gazdzinski et al., 2025), was administered to all mice via daily intraperitoneal injections for 3 days, which activated Cre recombinase in Cre+ mice, thereby deleting the Myrf gene in NG2-expressing cells in these mice (OPC-MyrfKO) and halting oligodendrogenesis. Cre− mice served as controls (OPC-MyrfKO-CTRL) with intact oligodendrogenesis. The mice were given supplemental food (DietGel 76A, Clear H2O, USA) beginning immediately after the final task session before tamoxifen and until 24 h after the last tamoxifen injection to decrease morbidity associated with the drug.

### 2.3 5 -Choice serial reaction time (5-CSRT) task

Mice were trained in 16 Bussey-Saksida touchscreen operant chambers (Campden Instruments, USA) following the protocol described below, adapted from the protocols described by Horner et al. (2013) and Mar et al. (2013) and with guidance from the Touchscreen Cognition website.^1^ One wall of the chamber consisted of a touchscreen on which an illuminated square stimulus was presented in one of five locations. The opposite wall of the chamber contained a reward port, in which strawberry milkshake (Neilson, Canada) was delivered following a correct response. Photographs and a graphical representation of the system and task are published in Mar et al. (2013). Data from one of the chambers (3 mice) was excluded from all analysis because there was an intermittent malfunction, making the data from this chamber unreliable.

#### 2.3.1 Food restriction

The mice were food-restricted beginning at 6–9 weeks of age and for the duration of the study to maintain a target weight of 80%–85% of their free-feeding weight. If food restriction began prior to 8 weeks of age, the target weight was increased by 1 g/week to account for developmental growth. Habituation and training began after 1–2 weeks of food restriction.

#### 2.3.2 Habituation and pre-training

The mice were habituated to the chambers and the milkshake over 2 days and then progressed through several stages of pre-training over the following 2–4 weeks in which they learned to associate touching the stimulus with the delivery of milkshake reward. Details about the stages are provided in the Supplementary material.

#### 2.3.3 Task-specific training

For this stage of the training, mice were required to initiate each trial by poking their head into the reward port, after which there was a 5-s delay before a stimulus would be presented on the screen. The mice were required to touch the stimulus window either while it was illuminated or during the 5-s limited hold period that followed to receive rewards. An incorrect response, an omitted trial, or a touch during the 5 s before stimulus presentation (premature touch), would end the trial and trigger the chamber light to come on and a buzzer to sound for 5 s. The stimulus duration was progressively reduced from 8 s to 4 s to 2 s. Mice were required to complete 40 correct trials within 60 min with an accuracy (# correct/(# correct + # incorrect + # omitted) × 100%) of at least 70% and fewer than 20% omitted trials on 3 out of 4 consecutive days to progress to the next stimulus duration. This stage of training lasted on average 5–6 weeks with the mice being trained 6 days/week. Mice that completed training earlier than others in their cohort were given weekly reminder sessions with the 2-s stimulus duration.

#### 2.3.4 Testing

At 8 days, 4 weeks, 8 weeks, and 12 weeks after the first mTBI procedure, the mice were tested on the task with stimulus durations of 0.8 s, 0.4 s, and 0.2 s. Prior to each of these probe trial series, the mice had three rebaselining sessions with the 2-s stimulus duration. Single rebaselining sessions were also done between each of the probe trials. Weekly reminder sessions with the 2-s stimulus duration were done between post-mTBI timepoints.

#### 2.3.5 Measures

The number of correct, incorrect, omitted and premature trials were counted and from this, accuracy (# correct/(# correct + # incorrect + # omitted) × 100%), choice accuracy (# correct/(# correct + incorrect) × 100%), % omitted trials and % premature trials were calculated. Correct response, incorrect response, and reward collection latencies were also recorded.

### 2.4 Repeated mild TBI

Following tamoxifen administration, the mice remained in their home cage for 2 days and then performed three rebaselining sessions with the 2-s stimulus duration. The average accuracy from these three sessions was considered when assigning the mice to the mTBI or sham groups such that the injury groups were balanced for task performance as well as genotype (Cre status) and sex. The mice were then subjected to three mTBI or sham procedures spaced 24 h apart. Under isoflurane anesthesia (4% for induction; 1%–2% for maintenance), the fur on the scalp was removed using depilatory cream (Nair, Church & Dwight Co., Inc., USA) and the mouse was positioned in a stereotaxic frame (Stoelting Co., USA) using zygomatic arch cuffs. An electromagnetic controlled cortical impact device (Impact One, Leica Biosystems, USA) was used to deliver an impact to the intact scalp centered at bregma with a 5-mm metal tip to a depth of 1.2 mm at a velocity of 2 m/s and with a dwell time of 0.2 s. Following the impact, the mouse was allowed to breathe oxygen for 1 min and was then transferred to a warm cage to recover from anesthesia before being returned to its home cage. Subcutaneous saline, as well as bupivacaine (0.125%) and xylocaine (0.1%) under the scalp, were administered before each procedure to maintain hydration and provide local analgesia, respectively, and sustained-release buprenorphine (1.2 mg/kg) was provided before the first and third procedures to provide systemic analgesia. The mice were given supplemental food following each mTBI procedure, as during tamoxifen administration.

### 2.5 Mild TBI and Myrf-cKO model validation

#### 2.5.1 Neurosilver staining and quantification

To confirm the presence of white matter damage following mTBI, axonal degeneration was visualized using the FD Neurosilver Kit II (FD Neurotechnologies Inc.). Briefly, 12 days after the final probe trial session, a subset of mice (*N* = 1/sex/genotype/injury group) were fixed via transcardial perfusion with heparinized phosphate buffered saline (PBS) followed by 4% paraformaldehyde (PFA) in PBS. The brains were left in the skulls and post-fixed overnight in 4% PFA before being transferred to PBS containing 0.02% sodium azide for storage at 4°C until further processing. In preparation for staining, the brains were dissected, cryoprotected in 15% sucrose for 1 day followed by 2 days in 30% sucrose in PBS before being cryosectioned at 40 μm into PBS-containing wells. Neurosilver staining was performed following the protocol provided with the kit.

Microscopy images of Neurosilver-stained sections of the corpus callosum at the midline (∼1.8 mm posterior to bregma) were acquired at 20× magnification on a Nikon Eclipse Ni-U microscope (1 image/sample). Staining quantification was performed using Fiji (ImageJ2 2.16.0/1.54n) (Schneider et al., 2012). The images were converted to 8-bit grayscale and background subtraction was performed using the sliding paraboloid method with a rolling ball radius of 10 pixels and a threshold defining positive stain was obtained using the Triangle method on each image. The area fraction of positive stain was measured in manually drawn ROIs of the corpus callosum.

#### 2.5.2 Electron microscopy

Corpus callosum samples were prepared for electron microscopy (EM), as described in detail previously (Gazdzinski et al., 2025), to characterize the effect of OPC-MyrfKO on white matter. Briefly, a subset of mice (*N* = 2/sex/genotype/injury group) were perfusion-fixed similarly to those prepared for histological staining, except that the fixative consisted of 2% PFA and 2.5% glutaraldehyde in 0.1 M sodium cacodylate buffer (EM fixative). The brains were dissected from the skulls immediately following the perfusion procedure and stored in 1% glutaraldehyde in sodium cacodylate buffer until being prepared for imaging. Corpus callosum samples were dissected (∼1 mm × 1 mm × 2 mm, approximately at bregma), post-fixed in 1% osmium tetroxide, dehydrated in a graded ethanol series, washed with propylene oxide and embedded in Quetol-Spurr resin. Blocks were cured overnight in a 60°C oven. Ultra-thin sections were cut on a Leica EM UC7 ultramicrotome, post-stained with 2% uranyl acetate and 3% lead citrate and then air dried at room temperature before viewing under EM. EM images (6–9 per sample) were acquired at 8000× magnification on a Hitachi HT7800 TEM, operated at 80 keV using a EMSIS Xarosa 20 MP CMOS camera. Myelinated axons were automatically segmented and counted on the EM images using AxonDeepSeg, as described previously (Zaimi et al., 2018; Gazdzinski et al., 2025).

### 2.6 Statistical analyses

Group comparisons in each of the 5-CSRT measures were made in the probe trial data (0.8 s, 0.4 s, and 0.2 s stimulus durations) using linear mixed effects models that included covariates for injury group, genotype, sex, stimulus duration, pre-mTBI performance, and timepoint post-mTBI, and random intercepts for mouse and cohort to account for repeated testing and correlations among mice tested together. The Satterthwaite approximation was used to estimate degrees of freedom for fixed effects. Interaction terms to test for injury group and genotype effects on change in measures over time (injury group × genotype × time; injury group × time; genotype × time) were also included in the models but only retained if the interaction term was significant at *p* < 0.05. Bonferroni correction was applied across the two primary performance measures reported (accuracy and premature touches) and separately across the three latency measures. Where a significant main effect or interaction was observed, separate linear models were run at each of the timepoints.

Similarly, group comparisons in myelinated axon density measured from EM images were made using a linear mixed effects model that included covariates for injury group, genotype, and sex, and random intercepts for mouse to account for correlations among images from the same animal. Group comparisons in the Neurosilver-stained area fraction in the corpus callosum were made using a linear model that included covariates for injury group, genotype, and sex. Statistical analyses were performed in R (4.4.0; RStudio 2024.09.1+394 for macOS) (R Core Team, 2022).

## 3 Results

### 3.1 Mild TBI and Myrf-cKO model validation

Confirming the occurrence of head impact, mTBI mice experienced a short period of apnea immediately post-impact (apnea duration following the first mTBI: mean = 8.7 s, SD = 8.2 s; data available in a subset of 26 mice) whereas all sham mice experienced no apnea. Evidence of axonal degeneration was observed in the corpus callosum of mTBI mice at ∼14 weeks post-injury (β = 4.32%, *p* = 0.041, Supplementary Figure 1), confirming persistent mTBI damage in a structure involved in complex cognitive function, including attention (Banich, 1998; Brown et al., 2020).

As expected, OPC-MyrfKO resulted in a lower density of myelinated axons in the corpus callosum of OPC-MyrfKO mice compared to OPC-MyrfKO-CTRL mice at 14 weeks post-tamoxifen administration (β = −0.098 axons/μm^2^, *p* = 0.038, Supplementary Figure 2). This aligns with previously published evidence that the OPC-MyrfKO model successfully blocks oligodendrogenesis in our hands (Supplementary Figure 1; Gazdzinski et al., 2025).

### 3.2 5 -CSRT task performance

The mice learned the 5-CSRT task and performed at a level above chance (1 in 5 = 20% choice accuracy) in post-mTBI probe trials. The evolution of accuracy and omissions over the course of the final training stage, as well as the total time required to complete the training for all mice is shown in Supplementary Figure 3. Choice accuracy decreased (β = −5.93% per 0.1 s decrease in stimulus duration, *p* < 2 × 10^–16^) and % omissions increased (β = 1.61% per 0.1 s decrease in stimulus duration, *p* < 2 × 10^–16^) with decreasing stimulus duration, confirming that attention was challenged as the stimulus duration was decreased (Supplementary Figure 4). Contrary to expectation, no effect of mTBI was observed on any of the 5-CSRT task measures at any of the timepoints or stimulus durations.

**FIGURE 2:**
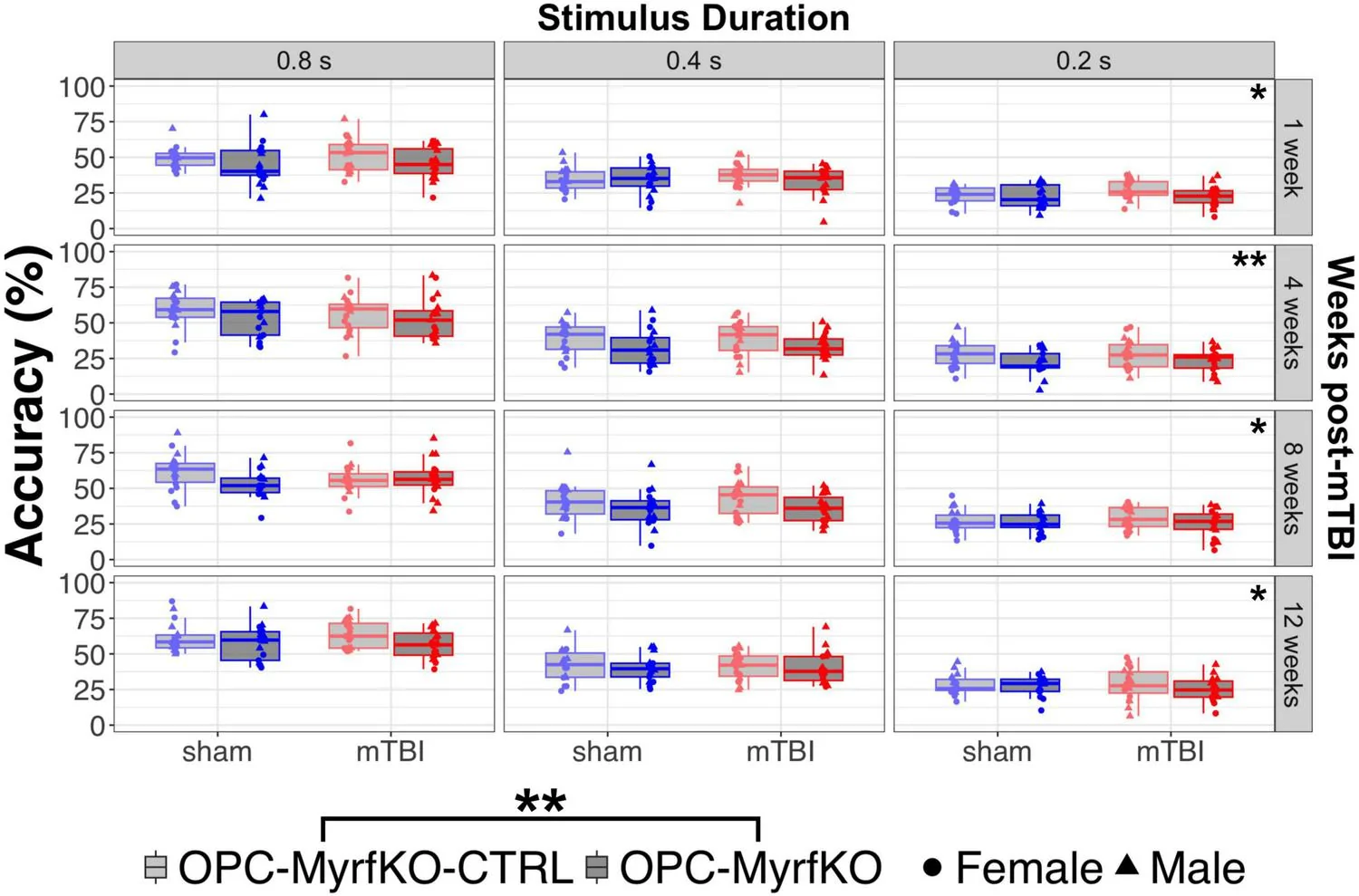
Accuracy on the 5-CSRT task in post-mTBI probe trials. OPC-MyrfKO mice had lower accuracy (β = –4.26%, p_Bonf_ = 0.003) across injury groups, stimulus durations and timepoints, as well as across injury groups and stimulus durations at each timepoint. No effect of mTBI was observed. *p < 0.05, **p < 0.01 main effect of genotype across injury groups, stimulus durations and timepoints and across injury groups and stimulus durations at individual timepoints where indicated.

In the probe trial sessions, OPC-MyrfKO mice omitted more trials (β = 3.9%, *p* = 0.022, Supplementary Figure 4) and had fewer correct trials (β = −1.95, *p* = 0.049), than OPC-MyrfKO-CTRL mice, resulting in lower accuracy across injury groups and probe trial stimulus durations at all timepoints (main effect of genotype across all timepoints: β = −4.26%, p_Bonf_ = 0.003; Figure 2), suggesting impaired attention in these mice.

**FIGURE 3:**
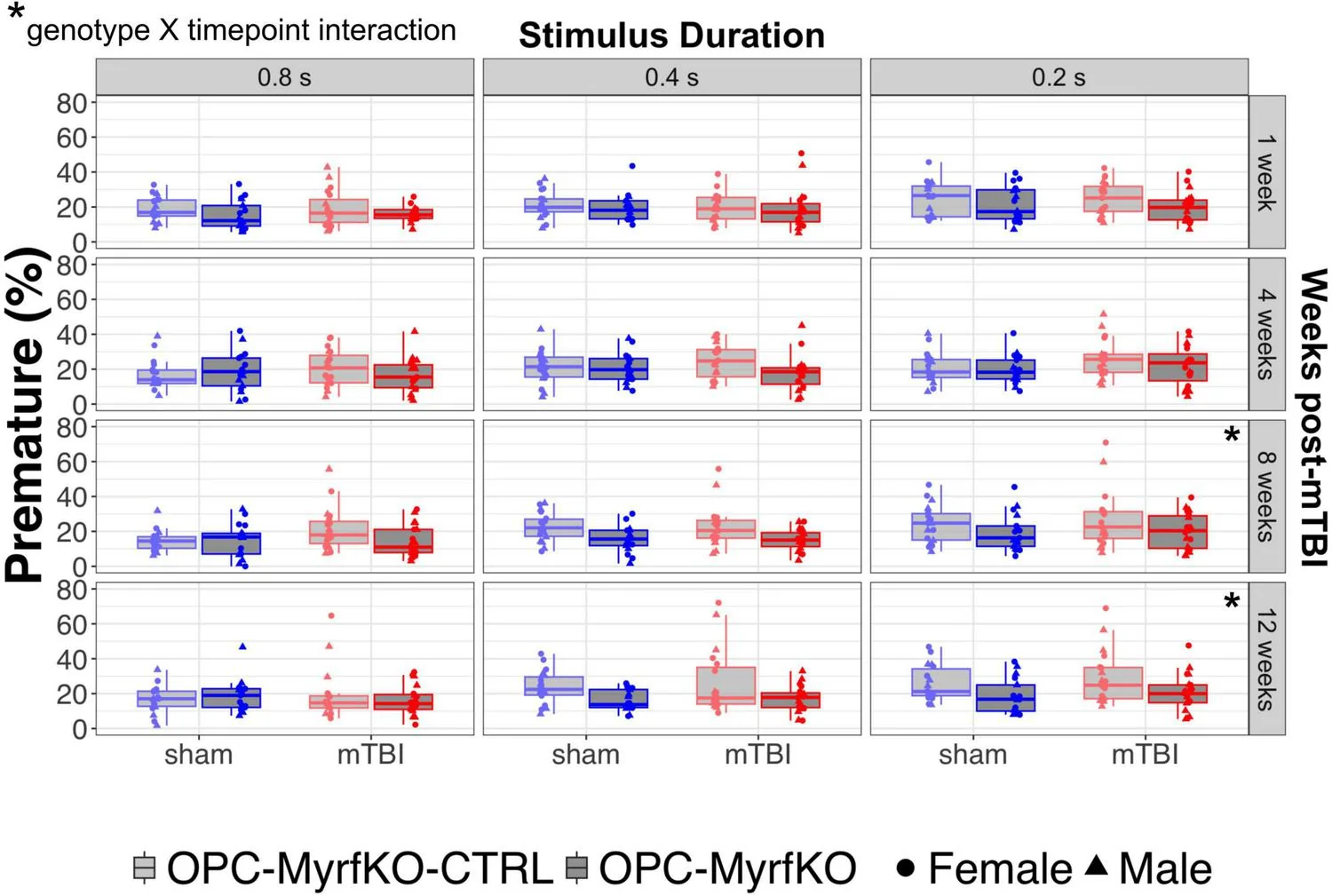
Premature trials on the 5-CSRT task in post-mTBI probe trials. There was a significant genotype × timepoint interaction in the proportion of premature trials (β = –0.30%, p_Bonf_ = 0.03) and OPC-MyrfKO mice had fewer premature trials than OPC-MyrfKO-CTRL mice at the 8- and 12-week timepoints. *p < 0.05 genotype × timepoint interaction and main effect of genotype across injury groups and stimulus durations at individual timepoints where indicated.

The inter-probe rebaselining sessions (2-s stimulus duration) provided a means to investigate whether the observed impairment was specific to the reduced stimulus duration sessions, in which attention was challenged. Accuracy was similar between OPC-MyrfKO and OPC-MyrfKO-CTRL mice in these rebaselining trials, however, OPC-MyrfKO mice had more omitted trials during these sessions (β = 1.3%, *p* = 0.041), indicating that these mice were more likely to omit trials even if they were unlikely to have missed the stimulus. This suggests that the OPC-MyrfKO mice may have been less motivated to perform the task (Supplementary Figure 5). A significant genotype-by-timepoint interaction in the proportion of premature trials was also observed (β = −0.30%, p_Bonf_ = 0.03), reflecting that by 8 weeks post-mTBI, OPC-MyrfKO mice had significantly fewer premature trials than OPC-Myrf-CTRL mice (β = −4.8%, *p* = 0.017 at 8 weeks and β = −5.19%, *p* = 0.027 at 12 weeks, Figure 3). While an increase in premature trials is usually interpreted as a lack of inhibitory control, the decrease observed here supports the interpretation that OPC-MyrfKO mice may have been less engaged by the task and therefore generally less likely to interact with the touchscreen. The widening difference in performance between the OPC-MyrfKO and OPC-MyrfKO-CTRL mice over time suggests that the impact of halted oligodendrogenesis may increase over time. The interpretation of a decreased level of motivation in the OPC-MyrfKO mice is further supported by the observation that these mice were slower to collect the reward (β = 0.15 s, p_Bonf_ = 0.014) compared to OPC-MyrfKO-CTRL mice (Supplementary Figure 6). Several published studies have provided evidence that OPC-MyrfKO mice have normal locomotor function (McKenzie et al., 2014; Shimizu et al., 2023). This is also supported by data from a separate study in our lab, in which OPC-MyrfKO mice had similar total alternations on the Y-maze out to 12 weeks (β = −1.81, *p* = 0.165) after tamoxifen administration compared to OPC-MyrfKO-CTRL mice (Supplementary Figure 7). Together, these results suggest the poorer task performance by OPC-MyrfKO mice is unlikely to be driven by locomotor deficit and that attention and motivation may both be impacted by disruption of adult oligodendrogenesis. A table of all statistical results is provided in the supplemental material (Supplementary Data Sheet 1).

## 4 Discussion

Difficulty with focus and attention is common after mTBI in humans (Stojanovski et al., 2021) and has been reported in several rodent model studies (Mychasiuk et al., 2015; Xu et al., 2021; Zhang et al., 2021; Vonder Haar et al., 2022). Given the role for myelin in supporting and tuning neural circuit function (Noori et al., 2020), we aimed to investigate how inhibiting oligodendrogenesis affects attention in mice under sham and repeated mTBI conditions, to test whether ongoing myelination contributes to recovery of attention capabilities following injury. Although we did not observe impaired attention following mTBI in this study, precluding the ability to test the planned hypothesis, halting adult oligodendrogenesis did produce modest broad impairment on task performance, providing some insight into the potential role of new myelin in attention and possibly motivation and complex task performance.

The lack of an effect of mTBI on attention measures in the 5-CSRT task suggests that the model used in the current study may be too mild or may not have the injury characteristics required to elicit measurable impairment in 5-CSRT task performance. A similar mouse model that delivered one mTBI rather than three impacts was previously characterized with transient behavioral impairment observed 3 days after injury specific to the Y-maze test sensitive to working memory and attention (Gazdzinski et al., 2020). The same single impact model demonstrated persistent structural differences and neuroinflammation in white matter (Gazdzinski et al., 2020). That said, it is not uncommon for animal models of mTBI (Shultz et al., 2012; To and Nasrallah, 2021), as well as human neuroimaging studies (Manning et al., 2017; Churchill et al., 2019), to demonstrate ongoing differences in the brain following mTBI with no detectable behavioral differences or symptoms. The absence of behavioral deficits despite the presence of structural damage may reflect functional compensation or structural redundancy, as has been demonstrated in both human and animal studies (McAllister et al., 2001; Wolf et al., 2017). Additionally, the mice may have learned the task so well that the implementation of the 5-CSRT task may not have sufficiently challenged damaged networks to elicit measurable deficits. Though previous rodent studies have reported attentional impairment post-mTBI using the 5-CSRT task, they, in general, involved a higher number of impacts (≥5), incorporated a higher degree of rotational acceleration, were conducted in rats, or trained the animals after the injury, in which case task performance may be confounded by learning challenges post-mTBI (Mychasiuk et al., 2015; Xu et al., 2021; Vonder Haar et al., 2022).

Despite the absence of an attentional deficit following mTBI, the results suggest a role for oligodendrogenesis in attention and task engagement. Accuracy was high with the 0.8 s stimulus duration and decreased in all groups with decreasing stimulus duration, confirming that the mice learned the task well and that attention was being challenged with the shorter stimulus durations. Mice with halted oligodendrogenesis had lower accuracy across injury groups and probe-trial stimulus durations at all timepoints and had fewer premature trials at the later timepoints. These mice were also slower to collect the rewards following correct touches. OPC-MyrfKO mice had similar accuracy as controls with the longer 2-s stimulus duration presented during the inter-probe trial rebaselining sessions, supporting the interpretation that attention is impaired when adult oligodendrogenesis is blocked. The higher omission rate and longer reward collection latency of these mice across injury groups, stimulus durations, and timepoints, and the lower rate of premature trials at the later timepoints, suggest that these mice may also be less motivated to perform the task and that the effect of oligodendrogenesis inhibition may broaden over time.

Neuronal activity promotes oligodendrogenesis and adaptive myelination (Gibson et al., 2014) and oligodendrocytes provide support to axons through functions unrelated to myelin (Duncan et al., 2021; Krämer-Albers and Werner, 2023; Simons et al., 2024). These functions allow oligodendrocytes to contribute to experience dependent fine-tuning of neural circuits important for learning and memory (McKenzie et al., 2014; Xiao et al., 2016; Bacmeister et al., 2020; Pan et al., 2020; Steadman et al., 2020; Munyeshyaka and Fields, 2022; Shimizu et al., 2023; Yalçın et al., 2024). Reduced myelin turnover and remodeling reduces circuit plasticity, potentially impairing network function required for cognition. Evidence for altered brain activity following the inhibition of oligodendrogenesis in a similar mouse model to the one used here has been recently demonstrated using electroencephalography (EEG) by Kaller et al. (2024).

Altered patterns of neural activity and myelination have been reported in attention deficit and hyperactivity disorder (Lin et al., 2024; Gao et al., 2025; Gholamipourbarogh et al., 2025; Hu et al., 2025; Mizuno et al., 2025) and reduced neural activity and myelin content have been observed in several conditions associated with anhedonia and lack of motivation, including social deprivation (Liu et al., 2012; Hong et al., 2023), chronic stress (Kokkosis et al., 2022), and major depressive disorder (Sacchet and Gotlib, 2017). Relevant to the results presented here, using similar mice, a recent study by Yalçın et al. (2024) demonstrated that active oligodendrogenesis is required for opioid-induced rewards learning using the conditioned place preference (CPP) test. Interestingly, this did not translate to general rewards behavior such that the mice displayed normal food-induced CPP, naloxone-induced aversion learning, and sucrose preference. While food reward is a component of the 5-CSRT paradigm, the task is more complex than Pavlovian conditioning and therefore likely relies on coordinated activity across a distinct neural network. The details of this network and the mechanisms underlying the role of oligodendrogenesis in maintaining its function remain open questions.

There are limitations that should be considered while interpreting the results from this study. First, a comprehensive characterization of brain pathology beyond the presence of axonal degeneration in the corpus callosum following mTBI was not included, therefore the extent of the injury is assumed based on previous work. Several studies using similar models, including some involving a single impact, have reported pathological changes in the white matter assessed with histopathology and/or magnetic resonance imaging that persisted for several weeks to months post-injury (Yu et al., 2017; Lyons et al., 2018; Gazdzinski et al., 2020). Second, while overt motor or sensory deficits were not observed in the OPC-MyrfKO mice and we provide evidence from a separate study that suggests that these mice have normal locomotor ability, a comprehensive assessment of motor and sensory function was not included, thus it is possible that subtle deficits in these domains contribute to the poorer task performance in these mice. It is also possible that these mice may be more sensitive than OPC-MyrfKO-CTRL mice to the prolonged food deprivation and/or age-related changes, which may contribute to the observed phenotype. Third, the inducible MyrfKO model relies on tamoxifen administration and Cre recombinase under the control of the NG2 promoter. Tamoxifen can have toxic side effects and while this is expected to be common across all groups in the study, it is possible that some mice are more susceptible to these effects than others. NG2 is expressed not only in OPCs, but also in other cells, including pericytes, thus it is likely that Cre recombinase activity was also induced in off-target cells. As Myrf is not expressed in these other cell populations, the Cre recombinase activity in these other cell populations is unlikely to have had a direct effect on gene expression in these cells, but other effects cannot be ruled out (McKenzie et al., 2014; Pan et al., 2020; He et al., 2024; Kaller et al., 2024; Xin et al., 2024; Gazdzinski et al., 2025).

In conclusion, difficulty with attention is common after mTBI and can affect many aspects of cognition and quality of life. Identification of therapeutic targets is urgently needed and given myelin’s role in neural circuit function and its vulnerability to injury, myelin plasticity remains a potential candidate. While we did not observe impaired attention on the 5-CSRT task following mTBI in this study, mice unable to generate new myelin showed reduced task accuracy, possibly influenced by motivational processes. These findings suggest that adult oligodendrogenesis supports attention and complex behavior. Future research may aim to optimize the injury model to better recapitulate the cognitive impairments observed in humans, including attention, or investigate the role of oligodendrogenesis in motivation with tests better suited to specifically measure this behavior.

## Data Availability

The raw data supporting the conclusions of this article will be made available by the authors, without undue reservation.

## Footnotes

1
https://touchscreencognition.org/

## Supplementary material

Supplementary Figure 1 Neurosilver staining in the corpus callosum at 14 weeks post-mTBI. (A) Representative images of Neurosilver staining in the corpus callosum of sham (left) and mTBI (right) mice. Axonal degeneration is visualized with black precipitate that is prominent in the mTBI image. The areas in the solid white bordered boxes are magnified views of the areas outlined in the hashed boxes. Scale bar = 100 m (B) Quantification of Neurosilver-stained area fraction in the corpus callosum showing increased staining in mTBI mice compared to shams (β = 4.32%, *p* = 0.041). **p* < 0.05.

Supplementary Figure 2 Quantification of myelinated axon density at 15 weeks post-tamoxifen administration. **(A)** Representative EM images of the corpus callosum of OPC-MyrfKO-CTRL (left) and OPC-MyrfKO (right) sham (top row) and mTBI (bottom row) mice. Scale bar = 1 μm **(B)** Quantification of the density of myelinated axons in the corpus callosum. There is a main effect of genotype such that OPC-MyrfKO mice have a lower density of myelinated axons than OPC-MyrfKO-CTRL mice across injury groups (β = -0.098 axons/μm2, *p* = 0.038). **p* < 0.05.

Supplementary Figure 3 Task-specific training. Evolution of accuracy **(A)** and omissions **(B)** over the course of the final 5-CSRT training stage (2-s stimulus duration). Each coloured line represents an individual mouse while the black fitted line represents a local polynomial regression fit to the data with the 95% confidence interval indicated by the shaded region. One mouse never reached criterion but was included in the study as it was not an outlier in testing trials. **(C)** Total time taken to complete the task-specific training, plotted by group. Each point represents the total for each mouse and was calculated as the sum of the number of days to reach criterion at each stage of the task-specific training (5-CSRT with 8 s, then 4 s, then 2 s stimulus duration). There were no group differences in the amount of time taken for the mice to complete the training.

Supplementary Figure 4 Choice accuracy **(A)** and omissions **(B)** on the 5-CSRT task in post-mTBI probe trials. Choice accuracy decreased and omissions increased with decreasing stimulus duration (*p* < 2 × 10^16^), confirming that attention was challenged with the shorter stimulus durations. OPC-MyrfKO mice had lower choice accuracy (β = –2.99%, *p* = 0.13) and more omissions (β = 3.9%, *p* = 0.022) than OPC-MyrfKO-CTRL mice across injury groups, stimulus durations and timepoints. No effect of mTBI was observed in these measures. For improved visualization, 1 data point in (A) and 3 data points in (B) are outside the y axis limits and not shown. All data points were included in the statistical models. **p* < 0.05 main effect of genotype across injury groups, stimulus durations and timepoints and across injury groups and stimulus durations at individual timepoints where indicated.

Supplementary Figure 5 Choice accuracy **(A)** and omissions **(B)** on the 5-CSRT task in inter-probe trial rebaselining sessions post-mTBI. OPC-MyrfKO mice had more omissions (β = 1.3%, *p* = 0.041) than OPC-MyrfKO-CTRL mice across injury groups and timepoints, while choice accuracy was similar across groups. No effect of mTBI was observed in these measures. **p* < 0.05 main effect of genotype across injury groups, rebaselining sessions and timepoints and across injury groups and rebaselining sessions at individual timepoints where indicated.

Supplementary Figure 6 Correct response **(A)**, incorrect response **(B)**, and reward collection **(C)** latencies. OPC-MyrfKO mice had longer reward collection latency (β = 0.15 s, p_Bonf_ = 0.014) compared to OPC-MyrfKO-CTRL. Incorrect response latency was longer than correct response latency (β = 0.7 s, *p* < 2 × 10^–16^). For improved visualization, 5 data points > 2.5 s in (C) are outside the y axis limits and not shown. All data points were included in the statistical models. **p* < 0.05, ***p* < 0.01 genotype X timepoint interaction or main effect of genotype across injury groups, stimulus durations and timepoints and across injury groups and stimulus durations at individual timepoints where indicated.

Supplementary Figure 7 Total number of alternations on the Y-maze in OPC-MyrfKO and OPC-MyrfKO-CTRL mice out to 12 weeks post-tamoxifen administration. These results are from a separate study that used the same mouse line and tamoxifen dosing as the current study. Mice were administered tamoxifen at 8 weeks of age and tested longitudinally on the Y-maze spontaneous alternation task at 1, 2, 6, and 12 weeks post-tamoxifen administration. OPC-MyrfKO mice had a similar number of total alternations as OPC-Myrf-CTRL mice, suggesting they explored the maze similarly and providing evidence of normal locomotor function in these mice.

Supplementary Table 1 Statistics table. Effects sizes (β estimates) and significance measures for the fixed effects variables in the linear mixed effects models run for each measure.
