# Supplementary material for: Inhibited oligodendrogenesis impairs attention and motivation in adult mice regardless of repeated mild traumatic brain injury
Source: Front Behav Neurosci. 2026 Aug 31;20:1862945. doi: 10.3389/fnbeh.2026.1862945 (PMC13572726; doi:10.3389/fnbeh.2026.1862945)
Supplement: Supplementary file 1 [file Data_Sheet_1.pdf]

Table S1: STATISTICS SUMMARY

Linear mixed effects models (5-CSRT measures):
3-way interaction model: ~ Injury_group + Timepoint + Genotype + Sex + Stimulus Duration + pre-mTBI_performance + Injury_groupXTimepoint + Injury_groupXGenotype + GenotypeXTimepoint + Injury_groupXTimepointXGenotype + (1 | Cohort) + (1 | Mouse)
interaction model: ~ Injury_group + Timepoint + Genotype + Sex + Stimulus Duration + pre-mTBI_performance + Injury_groupXTimepoint + GenotypeXTimepoint + (1 | Cohort) + (1 | Mouse)
covariate model: ~ Injury_group + Timepoint + Genotype + Sex + Stimulus Duration + pre-mTBI_performance + (1 | Cohort) + (1 | Mouse)
individual timepoints: ~ Injury_group + Genotype + Sex + Stimulus Duration + pre-mTBI_performance + (1 | Cohort) + (1 | Mouse)

Linear model (Neurosilver staining):
~ Injury_group + Genotype + Sex

Linear mixed effects model (myelinated axon density):
~ Injury_group + Genotype + Sex + (1 | Mouse)

Linear mixed effects models (Y-maze):
interaction model: ~ Timepoint + Genotype + Sex + GenotypeXTimepoint + (1 | Mouse)
covariate model: ~ Timepoint + Genotype + Sex + (1 | Mouse)

Linear mixed effects models (training duration):
~ Injury_group + Genotype + Sex + (1 | Cohort)

					Test Statistics				Significance
**Figure**	**Outcome Measure**	**Model**	**Timepoint**	**Factors**	**Estimate (β)**	**Std Error**	**df**	**t-value**	**p-value**
2	Accuracy (%) probe trials	linear mixed effects 3-way interaction model	all	Injury group (mTBI vs sham)	0.021	0.022	135.800	0.986	0.326
				Timepoint (weeks)	0.007	0.001	798.000	4.671	0.000 ***
				Genotype (OPC-MyrfKO vs OPC-MyrfKO-CTRL)	-0.036	0.022	137.100	-1.633	0.105
				Sex (M vs F)	0.050	0.014	66.240	3.659	0.001 ***
				Stimulus duration (s)	0.478	0.012	798.000	40.687	<2e-16 ***
				pre-mTBI accuracy (%)	0.244	0.090	64.920	2.720	0.008 **
				Injury group X Timepoint interaction	-0.002	0.002	798.000	-0.877	0.380
				Injury group X Genotype interaction	-0.021	0.031	135.200	-0.690	0.492
				Genotype X Timepoint interaction	0.000	0.002	798.000	0.110	0.912
				Injury group X Timepoint X Genotype interaction	0.001	0.003	798.000	0.368	0.713
		linear mixed effects interaction model	all	Injury group (mTBI vs sham)	0.011	0.015	138.700	0.706	0.481
				Timepoint (weeks)	0.006	0.001	799.000	5.193	0.000 ***
				Genotype (OPC-MyrfKO vs OPC-MyrfKO-CTRL)	-0.047	0.015	140.300	-3.066	0.003 **
				Sex (M vs F)	0.050	0.014	67.080	3.643	0.001 ***
				Stimulus duration (s)	0.478	0.012	799.000	40.709	<2e-16 ***
				pre-mTBI accuracy (%)	0.241	0.089	65.980	2.702	0.009 **
				Injury group X Timepoint interaction	-0.001	0.001	799.000	-0.870	0.385
				Genotype X Timepoint interaction	0.001	0.001	799.000	0.543	0.588
		linear mixed effects covariate model	all	Injury group (mTBI vs sham)	0.003	0.012	62.770	0.249	0.804
				Timepoint (weeks)	0.006	0.001	801.000	8.672	<2e-16 ***
				Genotype (OPC-MyrfKO vs OPC-MyrfKO-CTRL)	-0.043	0.013	64.810	-3.359	0.001 **
				Sex (M vs F)	0.050	0.014	67.080	3.643	0.001 ***
				Stimulus duration (s)	0.478	0.012	801.000	40.734	<2e-16 ***
				pre-mTBI accuracy (%)	0.241	0.089	65.980	2.702	0.009 **
			1 week	Injury group (mTBI vs sham)	0.015	0.015	68.000	0.970	0.335
				Genotype (OPC-MyrfKO vs OPC-MyrfKO-CTRL)	-0.039	0.015	68.000	-2.565	0.013 *
				Sex (M vs F)	0.026	0.015	68.000	1.704	0.093 .
				Stimulus duration (s)	0.387	0.021	145.000	18.380	<2e-16 ***
				pre-mTBI accuracy (%)	0.302	0.106	68.000	2.857	0.006 **
			4 weeks	Injury group (mTBI vs sham)	-0.003	0.019	62.820	-0.171	0.865
				Genotype (OPC-MyrfKO vs OPC-MyrfKO-CTRL)	-0.053	0.019	65.323	-2.768	0.007 **
				Sex (M vs F)	0.050	0.020	64.551	2.470	0.016 *
				Stimulus duration (s)	0.500	0.023	145.000	21.669	<2e-16 ***
				pre-mTBI accuracy (%)	0.245	0.133	66.614	1.834	0.071 .
			8 weeks	Injury group (mTBI vs sham)	0.004	0.018	64.193	0.254	0.800
				Genotype (OPC-MyrfKO vs OPC-MyrfKO-CTRL)	-0.045	0.018	66.047	-2.532	0.014 *
				Sex (M vs F)	0.061	0.019	65.524	3.191	0.002 **

				Stimulus duration (s)	0.494	0.022	145.000	22.196	<2e-16	***
				pre-mTBI accuracy (%)	0.235	0.126	66.991	1.870	0.066	.
			12 weeks	Injury group (mTBI vs sham)	-0.005	0.018	64.117	-0.278	0.782	
				Genotype (OPC-MyrfKO vs OPC-MyrfKO-CTRL)	-0.037	0.018	67.292	-2.023	0.047	*
				Sex (M vs F)	0.056	0.018	47.430	3.011	0.004	**
				Stimulus duration (s)	0.530	0.019	145.000	28.186	<2e-16	***
				pre-mTBI accuracy (%)	0.154	0.127	67.989	1.215	0.229	
-	# Correct trials probe trials	linear mixed effects 3-way interaction model	all	Injury group (mTBI vs sham)	2.318	1.672	134.142	1.386	0.168	
				Timepoint (weeks)	0.162	0.111	798.002	1.453	0.147	
				Genotype (OPC-MyrfKO vs OPC-MyrfKO-CTRL)	-3.263	1.725	136.915	-1.892	0.061	.
				Sex (M vs F)	1.489	1.072	52.071	1.388	0.171	
				Stimulus duration (s)	19.338	0.920	798.002	21.029	<2e-16	***
				pre-mTBI correct trials	0.934	0.523	66.939	1.785	0.079	.
				Injury group X Timepoint interaction	-0.135	0.156	798.002	-0.869	0.385	
				Injury group X Genotype interaction	0.216	2.385	133.110	0.090	0.928	
				Genotype X Timepoint interaction	0.223	0.160	798.002	1.392	0.164	
				Injury group X Timepoint X Genotype interaction	-0.060	0.222	798.002	-0.269	0.788	
		linear mixed effects interaction model	all	Injury group (mTBI vs sham)	2.425	1.185	137.319	2.046	0.043	*
				Timepoint (weeks)	0.177	0.096	799.000	1.838	0.066	.
				Genotype (OPC-MyrfKO vs OPC-MyrfKO-CTRL)	-3.149	1.194	142.971	-2.638	0.009	**
				Sex (M vs F)	1.488	1.063	53.388	1.399	0.167	
				Stimulus duration (s)	19.338	0.919	799.000	21.041	<2e-16	***
				pre-mTBI correct trials	0.936	0.519	67.943	1.802	0.076	.
				Injury group X Timepoint interaction	-0.165	0.111	799.000	-1.486	0.138	
				Genotype X Timepoint interaction	0.192	0.111	799.000	1.732	0.084	.
		linear mixed effects covariate model	all	Injury group (mTBI vs sham)	1.397	0.962	60.891	1.452	0.152	
				Timepoint (weeks)	0.186	0.055	801.000	3.353	0.001	***
				Genotype (OPC-MyrfKO vs OPC-MyrfKO-CTRL)	-1.952	0.974	64.471	-2.005	0.049	*
				Sex (M vs F)	1.488	1.063	53.388	1.399	0.167	
				Stimulus duration (s)	19.338	0.921	801.000	21.000	<2e-16	***
				pre-mTBI correct trials	0.936	0.519	67.944	1.802	0.076	.
			1 week	Injury group (mTBI vs sham)	2.240	1.299	62.957	1.725	0.089	.
				Genotype (OPC-MyrfKO vs OPC-MyrfKO-CTRL)	-2.980	1.306	66.681	-2.282	0.026	*
				Sex (M vs F)	0.074	1.362	36.675	0.054	0.957	
				Stimulus duration (s)	18.317	1.797	145.000	10.194	<2e-16	***
				pre-mTBI correct trials	2.065	0.686	65.694	3.009	0.004	**
			4 weeks	Injury group (mTBI vs sham)	1.936	1.439	61.101	1.345	0.184	
				Genotype (OPC-MyrfKO vs OPC-MyrfKO-CTRL)	-2.556	1.456	64.630	-1.756	0.084	.
				Sex (M vs F)	2.028	1.588	53.104	1.277	0.207	
				Stimulus duration (s)	20.802	1.870	145.000	11.124	<2e-16	***
				pre-mTBI correct trials	0.865	0.776	67.922	1.114	0.269	
			8 weeks	Injury group (mTBI vs sham)	0.890	1.267	63.282	0.702	0.485	
				Genotype (OPC-MyrfKO vs OPC-MyrfKO-CTRL)	-1.434	1.282	65.758	-1.119	0.267	
				Sex (M vs F)	2.428	1.397	57.041	1.738	0.088	.
				Stimulus duration (s)	18.738	1.648	145.001	11.371	<2e-16	***
				pre-mTBI correct trials	0.803	0.683	67.937	1.175	0.244	
			12 weeks	Injury group (mTBI vs sham)	0.474	1.175	63.537	0.403	0.688	
				Genotype (OPC-MyrfKO vs OPC-MyrfKO-CTRL)	-0.745	1.190	65.666	-0.626	0.533	
				Sex (M vs F)	1.161	1.308	60.788	0.888	0.378	
				Stimulus duration (s)	19.496	1.694	145.000	11.512	<2e-16	***
				pre-mTBI correct trials	0.063	0.636	68.000	0.099	0.921	
-	# Incorrect trials probe trials	linear mixed effects 3-way interaction model	all	Injury group (mTBI vs sham)	1.525	2.064	138.880	0.739	0.461	
				Timepoint (weeks)	-0.317	0.138	798.000	-2.306	0.021	*
				Genotype (OPC-MyrfKO vs OPC-MyrfKO-CTRL)	-1.244	2.128	140.683	-0.585	0.560	
				Sex (M vs F)	0.682	1.311	62.014	0.520	0.605	
				Stimulus duration (s)	-38.026	1.136	798.000	-33.464	<2e-16	***
				pre-mTBI incorrect trials	0.909	0.309	66.991	2.939	0.005	**
				Injury group X Timepoint interaction	-0.182	0.192	798.000	-0.948	0.344	
				Injury group X Genotype interaction	1.520	2.945	138.153	0.516	0.607	

				Genotype X Timepoint interaction	0.302	0.198	798.000	1.528	0.127	
				Injury group X Timepoint X Genotype interaction	-0.142	0.274	798.000	-0.517	0.605	
		linear mixed effects interaction model	all	Injury group (mTBI vs sham)	2.276	1.462	142.377	1.557	0.122	
				Timepoint (weeks)	-0.282	0.119	799.000	-2.368	0.018	*
				Genotype (OPC-MyrfKO vs OPC-MyrfKO-CTRL)	-0.457	1.475	144.312	-0.309	0.757	
				Sex (M vs F)	0.725	1.300	63.382	0.558	0.579	
				Stimulus duration (s)	-38.026	1.136	799.000	-33.479	<2e-16	***
				pre-mTBI incorrect trials	0.904	0.307	67.997	2.950	0.004	**
				Injury group X Timepoint interaction	-0.252	0.137	799.000	-1.841	0.066	.
				Genotype X Timepoint interaction	0.228	0.137	799.000	1.669	0.096	.
		linear mixed effects covariate model	all	Injury group (mTBI vs sham)	0.702	1.186	62.926	0.592	0.556	
				Timepoint (weeks)	-0.300	0.069	801.000	-4.382	0.000	***
				Genotype (OPC-MyrfKO vs OPC-MyrfKO-CTRL)	0.969	1.203	65.064	0.806	0.423	
				Sex (M vs F)	0.725	1.300	63.383	0.558	0.579	
				Stimulus duration (s)	-38.026	1.139	801.000	-33.394	<2e-16	***
				pre-mTBI incorrect trials	0.904	0.306	67.997	2.950	0.004	**
3	Premature trials (%) probe trials	linear mixed effects 3-way interaction model	all	Injury group (mTBI vs sham)	0.011	0.024	102.600	0.433	0.666	
				Timepoint (weeks)	0.001	0.001	798.000	0.489	0.625	
				Genotype (OPC-MyrfKO vs OPC-MyrfKO-CTRL)	-0.012	0.025	102.700	-0.471	0.639	
				Sex (M vs F)	-0.027	0.016	67.000	-1.710	0.092	.
				Stimulus duration (s)	-0.088	0.010	798.000	-8.637	<2e-16	***
				pre-mTBI premature trials	0.345	0.112	67.000	3.091	0.003	**
				Injury group X Timepoint interaction	0.002	0.002	798.000	1.250	0.212	
				Injury group X Genotype interaction	-0.010	0.035	102.400	-0.280	0.780	
				Genotype X Timepoint interaction	-0.002	0.002	798.000	-1.323	0.186	
				Injury group X Timepoint X Genotype interaction	-0.001	0.002	798.000	-0.516	0.606	
		linear mixed effects interaction model	all	Injury group (mTBI vs sham)	0.006	0.017	104.200	0.335	0.738	
				Timepoint (weeks)	0.001	0.001	799.000	0.867	0.386	
				Genotype (OPC-MyrfKO vs OPC-MyrfKO-CTRL)	-0.017	0.017	104.000	-0.967	0.336	
				Sex (M vs F)	-0.027	0.016	68.000	-1.761	0.083	.
				Stimulus duration (s)	-0.088	0.010	799.000	-8.641	<2e-16	***
				pre-mTBI premature trials	0.349	0.111	68.000	3.141	0.002	**
				Injury group X Timepoint interaction	0.002	0.001	799.000	1.247	0.213	
				Genotype X Timepoint interaction	-0.003	0.001	799.000	-2.450	0.014	*
		linear mixed effects covariate model	1 week	Injury group (mTBI vs sham)	0.000	0.014	64.810	0.033	0.974	
				Genotype (OPC-MyrfKO vs OPC-MyrfKO-CTRL)	-0.023	0.014	67.080	-1.584	0.118	
				Sex (M vs F)	-0.035	0.015	52.350	-2.400	0.020	*
				Stimulus duration (s)	-0.090	0.018	145.000	-4.916	0.000	***
				pre-mTBI premature trials	0.381	0.103	66.770	3.702	0.000	***
			4 weeks	Injury group (mTBI vs sham)	0.021	0.020	64.791	1.079	0.285	
				Genotype (OPC-MyrfKO vs OPC-MyrfKO-CTRL)	-0.019	0.020	67.089	-0.986	0.328	
				Sex (M vs F)	-0.035	0.020	51.948	-1.718	0.092	.
				Stimulus duration (s)	-0.065	0.015	145.000	-4.283	0.000	***
				pre-mTBI premature trials	0.302	0.140	66.777	2.148	0.035	*
			8 weeks	Injury group (mTBI vs sham)	0.022	0.020	68.000	1.099	0.276	
				Genotype (OPC-MyrfKO vs OPC-MyrfKO-CTRL)	-0.048	0.020	68.000	-2.459	0.017	*
				Sex (M vs F)	-0.023	0.020	68.000	-1.190	0.238	
				Stimulus duration (s)	-0.095	0.018	145.000	-5.357	0.000	***
				pre-mTBI premature trials	0.424	0.140	68.000	3.033	0.003	**
			12 weeks	Injury group (mTBI vs sham)	0.019	0.023	68.000	0.808	0.422	
				Genotype (OPC-MyrfKO vs OPC-MyrfKO-CTRL)	-0.052	0.023	68.000	-2.256	0.027	*
				Sex (M vs F)	-0.015	0.023	68.000	-0.637	0.526	
				Stimulus duration (s)	-0.100	0.018	145.000	-5.519	0.000	***
				pre-mTBI premature trials	0.292	0.164	68.000	1.781	0.080	.
S1	Neurosilver staining (% area)	linear model	final (14 weeks post-mTBI)	Injury group (mTBI vs sham)	4.316	1.577	5.000	2.736	0.041	*
				Genotype (OPC-MyrfKO vs OPC-MyrfKO-CTRL)	0.498	1.577	5.000	0.316	0.765	
S2	Density of myelinated axons	linear mixed effects covariate model	final (15 weeks post-tamoxifen)	Injury group (mTBI vs sham)	-2.790	3.269	10.897	-0.853	0.412	
				Genotype (OPC-MyrfKO vs OPC-MyrfKO-CTRL)	-7.691	3.263	10.885	-2.357	0.038	*
				Sex (M vs F)	-1.184	3.266	10.871	-0.363	0.724	

S3	Training data	linear mixed effects covariate model	task-specific training stages (5-CSRT 8s-4s-2s stimulus duration)	Injury group (mTBI vs sham)	-0.753	1.242	64.843	-0.606	0.547	
				Genotype (OPC-MyrfKO vs OPC-MyrfKO-CTRL)	-0.365	1.262	66.173	-0.289	0.773	
				Sex (M vs F)	-0.592	1.367	68.705	-0.433	0.666	
S4	Choice accuracy (%) probe trials	linear mixed effects 3-way interaction model	all	Injury group (mTBI vs sham)	0.006	0.020	146.200	0.282	0.779	
				Timepoint (weeks)	0.005	0.001	798.000	3.293	0.001	**
				Genotype (OPC-MyrfKO vs OPC-MyrfKO-CTRL)	-0.021	0.021	147.600	-1.024	0.308	
				Sex (M vs F)	0.014	0.013	62.070	1.133	0.262	
				Stimulus duration (s)	0.593	0.011	798.000	52.453	<2e-16	***
				pre-mTBI choice accuracy	0.548	0.154	66.920	3.559	0.001	***
				Injury group X Timepoint interaction	0.000	0.002	798.000	0.238	0.812	
				Injury group X Genotype interaction	-0.013	0.029	145.600	-0.445	0.657	
				Genotype X Timepoint interaction	-0.001	0.002	798.000	-0.378	0.705	
				Injury group X Timepoint X Genotype interaction	0.001	0.003	798.000	0.313	0.754	
		linear mixed effects interaction model	all	Injury group (mTBI vs sham)	-0.001	0.014	149.700	-0.043	0.966	
				Timepoint (weeks)	0.004	0.001	799.000	3.630	0.000	***
				Genotype (OPC-MyrfKO vs OPC-MyrfKO-CTRL)	-0.028	0.014	150.800	-1.937	0.055	.
				Sex (M vs F)	0.014	0.013	63.060	1.117	0.268	
				Stimulus duration (s)	0.593	0.011	799.000	52.482	<2e-16	***
				pre-mTBI choice accuracy	0.545	0.153	67.920	3.571	0.001	***
				Injury group X Timepoint interaction	0.001	0.001	799.000	0.643	0.520	
				Genotype X Timepoint interaction	0.000	0.001	799.000	-0.220	0.826	
		linear mixed effects covariate model	all	Injury group (mTBI vs sham)	0.005	0.012	64.510	0.420	0.676	
				Timepoint (weeks)	0.005	0.001	801.000	6.778	0.000	***
				Genotype (OPC-MyrfKO vs OPC-MyrfKO-CTRL)	-0.030	0.012	66.190	-2.553	0.013	*
				Sex (M vs F)	0.014	0.013	63.060	1.117	0.268	
				Stimulus duration (s)	0.593	0.011	801.000	52.533	<2e-16	***
				pre-mTBI choice accuracy	0.545	0.153	67.930	3.571	0.001	***
			1 week	Injury group (mTBI vs sham)	0.001	0.016	64.730	0.035	0.972	
				Genotype (OPC-MyrfKO vs OPC-MyrfKO-CTRL)	-0.027	0.016	66.430	-1.683	0.097	.
				Sex (M vs F)	0.006	0.017	61.770	0.369	0.713	
				Stimulus duration (s)	0.528	0.021	145.000	25.541	<2e-16	***
				pre-mTBI choice accuracy	0.750	0.205	67.820	3.664	0.000	***
			4 weeks	Injury group (mTBI vs sham)	0.002	0.017	64.005	0.093	0.926	
				Genotype (OPC-MyrfKO vs OPC-MyrfKO-CTRL)	-0.030	0.017	65.803	-1.817	0.074	.
				Sex (M vs F)	0.015	0.018	63.620	0.844	0.402	
				Stimulus duration (s)	0.605	0.022	145.000	27.997	<2e-16	***
				pre-mTBI choice accuracy	0.659	0.219	67.972	3.015	0.004	**
			8 weeks	Injury group (mTBI vs sham)	0.010	0.016	65.166	0.651	0.518	
				Genotype (OPC-MyrfKO vs OPC-MyrfKO-CTRL)	-0.035	0.016	66.999	-2.187	0.032	*
				Sex (M vs F)	0.021	0.017	56.596	1.237	0.221	
				Stimulus duration (s)	0.624	0.021	145.000	29.893	<2e-16	***
				pre-mTBI choice accuracy	0.538	0.207	67.241	2.602	0.011	*
			12 weeks	Injury group (mTBI vs sham)	0.007	0.016	68.000	0.455	0.651	
				Genotype (OPC-MyrfKO vs OPC-MyrfKO-CTRL)	-0.029	0.016	68.000	-1.845	0.069	.
				Sex (M vs F)	0.015	0.016	68.000	0.932	0.354	
				Stimulus duration (s)	0.615	0.020	145.000	30.764	<2e-16	***
				pre-mTBI choice accuracy	0.205	0.201	68.000	1.021	0.311	
S4	Omissions (%) probe trials	linear mixed effects 3-way interaction model	all	Injury group (mTBI vs sham)	-0.036	0.028	125.900	-1.277	0.204	
				Timepoint (weeks)	-0.005	0.002	798.000	-2.969	0.003	**
				Genotype (OPC-MyrfKO vs OPC-MyrfKO-CTRL)	0.041	0.029	126.500	1.413	0.160	
				Sex (M vs F)	-0.073	0.018	66.920	-3.979	0.000	***
				Stimulus duration (s)	-0.161	0.015	798.000	-10.993	<2e-16	***
				pre-mTBI omissions	0.260	0.129	64.250	2.021	0.047	*
				Injury group X Timepoint interaction	0.003	0.002	798.000	1.401	0.161	
				Injury group X Genotype interaction	0.027	0.040	125.400	0.687	0.493	
				Genotype X Timepoint interaction	-0.002	0.003	798.000	-0.835	0.404	
				Injury group X Timepoint X Genotype interaction	-0.001	0.004	798.000	-0.207	0.836	
		linear mixed effects interaction model	all	Injury group (mTBI vs sham)	-0.022	0.020	128.209	-1.116	0.266	
				Timepoint (weeks)	-0.005	0.002	799.000	-3.317	0.001	***

				Genotype (OPC-MyrfKO vs OPC-MyrfKO-CTRL)	0.055	0.020	129.142	2.738	0.007	**
				Sex (M vs F)	-0.072	0.018	67.846	-3.951	0.000	***
				Stimulus duration (s)	-0.161	0.015	799.000	-11.000	<2e-16	***
				pre-mTBI omissions	0.258	0.128	65.341	2.009	0.049	*
				Injury group X Timepoint interaction	0.003	0.002	799.000	1.765	0.078	.
				Genotype X Timepoint interaction	-0.003	0.002	799.000	-1.423	0.155	
		linear mixed effects covariate model	all	Injury group (mTBI vs sham)	-0.003	0.017	62.310	-0.164	0.870	
				Timepoint (weeks)	-0.005	0.001	801.000	-5.328	0.000	***
				Genotype (OPC-MyrfKO vs OPC-MyrfKO-CTRL)	0.039	0.017	64.110	2.342	0.022	*
				Sex (M vs F)	-0.072	0.018	67.850	-3.951	0.000	***
				Stimulus duration (s)	-0.161	0.015	801.000	-10.979	<2e-16	***
				pre-mTBI omissions	0.258	0.128	65.340	2.009	0.049	*
			1 week	Injury group (mTBI vs sham)	-0.027	0.022	62.924	-1.267	0.210	
				Genotype (OPC-MyrfKO vs OPC-MyrfKO-CTRL)	0.046	0.022	65.111	2.088	0.041	*
				Sex (M vs F)	-0.051	0.023	66.044	-2.181	0.033	*
				Stimulus duration (s)	-0.105	0.028	145.000	-3.769	0.000	***
				pre-mTBI omissions	0.384	0.166	66.338	2.309	0.024	*
			4 weeks	Injury group (mTBI vs sham)	0.003	0.024	62.382	0.136	0.893	
				Genotype (OPC-MyrfKO vs OPC-MyrfKO-CTRL)	0.054	0.025	65.567	2.169	0.034	*
				Sex (M vs F)	-0.060	0.026	60.076	-2.293	0.025	*
				Stimulus duration (s)	-0.202	0.030	145.000	-6.737	0.000	***
				pre-mTBI omissions	0.274	0.188	66.927	1.458	0.150	
			8 weeks	Injury group (mTBI vs sham)	0.001	0.022	63.100	0.027	0.979	
				Genotype (OPC-MyrfKO vs OPC-MyrfKO-CTRL)	0.035	0.022	65.220	1.602	0.114	
				Sex (M vs F)	-0.082	0.023	66.090	-3.491	0.001	***
				Stimulus duration (s)	-0.145	0.027	145.000	-5.398	0.000	***
				pre-mTBI omissions	0.178	0.167	66.400	1.066	0.290	
			12 weeks	Injury group (mTBI vs sham)	0.015	0.022	63.310	0.702	0.485	
				Genotype (OPC-MyrfKO vs OPC-MyrfKO-CTRL)	0.025	0.022	66.356	1.111	0.271	
				Sex (M vs F)	-0.080	0.023	57.220	-3.480	0.001	***
				Stimulus duration (s)	-0.191	0.023	145.000	-8.446	0.000	***
				pre-mTBI omissions	0.200	0.168	67.411	1.195	0.236	
S5	Choice accuracy (%) rebaselining sessions	linear mixed effects 3-way interaction model	all	Injury group (mTBI vs sham)	-0.010	0.013	291.600	-0.740	0.460	
				Timepoint (weeks)	0.004	0.001	506.000	3.789	0.000	***
				Genotype (OPC-MyrfKO vs OPC-MyrfKO-CTRL)	0.000	0.014	296.500	-0.019	0.985	
				Sex (M vs F)	-0.012	0.006	52.110	-1.872	0.067	.
				Session in timepoint (2nd vs 1st)	-0.019	0.005	506.000	-4.050	0.000	***
				pre-mTBI choice accuracy	0.477	0.080	66.010	5.990	0.000	***
				Injury group X Timepoint interaction	0.002	0.002	506.000	0.922	0.357	
				Injury group X Genotype interaction	0.010	0.019	290.000	0.534	0.593	
				Genotype X Timepoint interaction	-0.002	0.002	506.000	-1.465	0.144	
				Injury group X Timepoint X Genotype interaction	0.001	0.002	506.000	0.389	0.697	
		linear mixed effects interaction model	all	Injury group (mTBI vs sham)	-0.005	0.009	292.097	-0.514	0.608	
				Timepoint (weeks)	0.004	0.001	507.000	4.161	0.000	***
				Genotype (OPC-MyrfKO vs OPC-MyrfKO-CTRL)	0.005	0.010	298.553	0.535	0.593	
				Sex (M vs F)	-0.011	0.006	49.771	-1.762	0.084	.
				Session in timepoint (2nd vs 1st)	-0.019	0.005	507.000	-4.053	0.000	***
				pre-mTBI choice accuracy	0.484	0.080	66.551	6.074	0.000	***
				Injury group X Timepoint interaction	0.002	0.001	507.000	1.680	0.094	.
				Genotype X Timepoint interaction	-0.002	0.001	507.000	-1.712	0.088	.
		linear mixed effects covariate model	all	Injury group (mTBI vs sham)	0.007	0.006	63.500	1.186	0.240	
				Timepoint (weeks)	0.004	0.001	509.000	7.278	0.000	***
				Genotype (OPC-MyrfKO vs OPC-MyrfKO-CTRL)	-0.007	0.006	66.470	-1.176	0.244	
				Sex (M vs F)	-0.011	0.006	49.770	-1.762	0.084	.
				Session in timepoint (2nd vs 1st)	-0.019	0.005	509.000	-4.039	0.000	***
				pre-mTBI choice accuracy	0.484	0.080	66.550	6.074	0.000	***
S5	Omissions (%) rebaselining sessions	linear mixed effects 3-way interaction model	all	Injury group (mTBI vs sham)	-0.001	0.013	271.400	-0.100	0.920	
				Timepoint (weeks)	-0.001	0.001	506.000	-0.628	0.530	
				Genotype (OPC-MyrfKO vs OPC-MyrfKO-CTRL)	0.013	0.014	275.800	0.986	0.325	
				Sex (M vs F)	-0.004	0.006	52.590	-0.612	0.543	

				Stimulus duration (s)	-0.010	0.005	506.000	-2.240	0.026	*
				pre-mTBI omissions	0.166	0.048	66.920	3.482	0.001	***
				Injury group X Timepoint interaction	0.000	0.002	506.000	-0.050	0.960	
				Injury group X Genotype interaction	0.010	0.019	270.300	0.528	0.598	
				Genotype X Timepoint interaction	0.000	0.002	506.000	-0.293	0.770	
				Injury group X Timepoint X Genotype interaction	-0.001	0.002	506.000	-0.345	0.730	
		linear mixed effects interaction model	all	Injury group (mTBI vs sham)	0.004	0.009	277.400	0.380	0.704	
				Timepoint (weeks)	-0.001	0.001	507.000	-0.526	0.599	
				Genotype (OPC-MyrfKO vs OPC-MyrfKO-CTRL)	0.018	0.009	282.600	1.974	0.049	*
				Sex (M vs F)	-0.004	0.006	53.330	-0.592	0.556	
				Session in timepoint (2nd vs 1st)	-0.010	0.005	507.000	-2.242	0.025	*
				pre-mTBI omissions	0.165	0.047	67.920	3.494	0.001	***
				Injury group X Timepoint interaction	0.000	0.001	507.000	-0.410	0.682	
				Genotype X Timepoint interaction	-0.001	0.001	507.000	-0.784	0.434	
		linear mixed effects covariate model	all	Injury group (mTBI vs sham)	0.001	0.006	65.340	0.112	0.912	
				Timepoint (weeks)	-0.001	0.001	509.000	-2.119	0.035	*
				Genotype (OPC-MyrfKO vs OPC-MyrfKO-CTRL)	0.013	0.006	67.530	2.084	0.041	*
				Sex (M vs F)	-0.004	0.006	53.330	-0.592	0.556	
				Session in timepoint (2nd vs 1st)	-0.010	0.005	509.000	-2.245	0.025	*
				pre-mTBI omissions	0.165	0.047	67.920	3.494	0.001	***
			1 week	Injury group (mTBI vs sham)	-0.003	0.012	65.430	-0.231	0.818	
				Genotype (OPC-MyrfKO vs OPC-MyrfKO-CTRL)	0.008	0.012	67.533	0.709	0.480	
				Sex (M vs F)	-0.009	0.012	54.068	-0.723	0.473	
				Session in timepoint (2nd vs 1st)	-0.014	0.010	72.000	-1.431	0.157	
				pre-mTBI omissions	0.228	0.090	67.922	2.538	0.013	*
			4 weeks	Injury group (mTBI vs sham)	0.014	0.011	68.000	1.263	0.211	
				Genotype (OPC-MyrfKO vs OPC-MyrfKO-CTRL)	0.024	0.011	68.000	2.152	0.035	*
				Sex (M vs F)	-0.001	0.011	68.000	-0.052	0.959	
				Session in timepoint (2nd vs 1st)	-0.013	0.009	72.000	-1.540	0.128	
				pre-mTBI omissions	0.175	0.085	68.000	2.055	0.044	*
			8 weeks	Injury group (mTBI vs sham)	-0.009	0.008	68.000	-1.197	0.236	
				Genotype (OPC-MyrfKO vs OPC-MyrfKO-CTRL)	0.019	0.008	68.000	2.512	0.014	*
				Sex (M vs F)	-0.001	0.008	68.000	-0.178	0.859	
				Session in timepoint (2nd vs 1st)	-0.016	0.007	72.000	-2.341	0.022	*
				pre-mTBI omissions	0.097	0.057	68.000	1.699	0.094	.
			12 weeks	Injury group (mTBI vs sham)	0.001	0.011	62.705	0.092	0.927	
				Genotype (OPC-MyrfKO vs OPC-MyrfKO-CTRL)	-0.003	0.011	64.450	-0.234	0.816	
				Sex (M vs F)	0.001	0.012	67.764	0.094	0.925	
				Session in timepoint (2nd vs 1st)	0.002	0.009	72.000	0.181	0.857	
				pre-mTBI omissions	0.202	0.087	65.614	2.329	0.023	*
S6	Correct response latency (s)	linear mixed effects 3-way interaction model	all	Injury group (mTBI vs sham)	0.099	0.064	53.000	1.558	0.125	
				Timepoint (weeks)	-0.015	0.003	18860.000	-5.434	0.000	***
				Genotype (OPC-MyrfKO vs OPC-MyrfKO-CTRL)	0.052	0.070	55.750	0.740	0.463	
				Sex (M vs F)	-0.011	0.045	37.960	-0.256	0.800	
				Stimulus duration (s)	-0.036	0.022	18860.000	-1.617	0.106	
				pre-mTBI correct latency	0.536	0.098	41.740	5.469	0.000	***
				Injury group X Timepoint interaction	-0.010	0.004	18860.000	-2.841	0.005	**
				Injury group X Genotype interaction	-0.141	0.095	52.860	-1.482	0.144	
				Genotype X Timepoint interaction	0.003	0.004	18860.000	0.663	0.507	
				Injury group X Timepoint X Genotype interaction	0.016	0.005	18860.000	2.866	0.004	**
		linear mixed effects interaction model	all	Injury group (mTBI vs sham)	0.034	0.045	54.640	0.753	0.455	
				Timepoint (weeks)	-0.018	0.002	18860.000	-7.884	0.000	***
				Genotype (OPC-MyrfKO vs OPC-MyrfKO-CTRL)	-0.026	0.046	57.260	-0.577	0.566	
				Sex (M vs F)	-0.012	0.044	38.890	-0.261	0.795	
				Stimulus duration (s)	-0.036	0.022	18860.000	-1.607	0.108	
				pre-mTBI correct latency	0.524	0.094	42.600	5.583	0.000	***
				Injury group X Timepoint interaction	-0.003	0.003	18860.000	-1.219	0.223	
				Genotype X Timepoint interaction	0.011	0.003	18860.000	4.146	0.000	***
		linear mixed effects covariate model	1 week	Injury group (mTBI vs sham)	0.053	0.045	39.052	1.162	0.253	
				Genotype (OPC-MyrfKO vs OPC-MyrfKO-CTRL)	-0.061	0.047	40.067	-1.310	0.198	

				Sex (M vs F)	0.064	0.052	43.861	1.247	0.219	
				Stimulus duration (s)	-0.038	0.052	4708.322	-0.719	0.472	
				pre-mTBI correct latency	0.630	0.106	41.575	5.961	0.000	***
			4 weeks	Injury group (mTBI vs sham)	-0.024	0.055	42.962	-0.435	0.666	
				Genotype (OPC-MyrfKO vs OPC-MyrfKO-CTRL)	0.023	0.054	42.957	0.417	0.679	
				Sex (M vs F)	-0.026	0.055	42.561	-0.466	0.643	
				Stimulus duration (s)	-0.029	0.044	4497.397	-0.652	0.514	
				pre-mTBI correct latency	0.577	0.120	43.279	4.815	0.000	***
			8 weeks	Injury group (mTBI vs sham)	0.030	0.055	39.150	0.542	0.591	
				Genotype (OPC-MyrfKO vs OPC-MyrfKO-CTRL)	0.135	0.055	41.460	2.442	0.019	*
				Sex (M vs F)	-0.056	0.057	31.690	-0.996	0.327	
				Stimulus duration (s)	0.005	0.039	4749.000	0.117	0.907	
				pre-mTBI correct latency	0.472	0.122	41.310	3.880	0.000	***
			12 weeks	Injury group (mTBI vs sham)	0.002	0.062	41.130	0.038	0.969	
				Genotype (OPC-MyrfKO vs OPC-MyrfKO-CTRL)	0.085	0.062	42.740	1.366	0.179	
				Sex (M vs F)	-0.019	0.063	33.430	-0.304	0.763	
				Stimulus duration (s)	-0.093	0.042	4838.000	-2.235	0.025	*
				pre-mTBI correct latency	0.359	0.137	42.310	2.619	0.012	*
S6	Incorrect response latency (s)	linear mixed effects 3-way interaction model	all	Injury group (mTBI vs sham)	0.059	0.089	68.800	0.660	0.511	
				Timepoint (weeks)	-0.007	0.006	9649.000	-1.117	0.264	
				Genotype (OPC-MyrfKO vs OPC-MyrfKO-CTRL)	-0.101	0.095	73.650	-1.058	0.294	
				Sex (M vs F)	-0.134	0.055	33.360	-2.436	0.020	*
				Stimulus duration (s)	0.750	0.060	9792.000	12.444	<2e-16	***
				pre-mTBI incorrect latency	0.020	0.035	32.630	0.564	0.577	
				Injury group X Timepoint interaction	-0.005	0.008	9641.000	-0.621	0.535	
				Injury group X Genotype interaction	0.173	0.131	70.450	1.316	0.193	
				Genotype X Timepoint interaction	0.014	0.009	9701.000	1.628	0.104	
				Injury group X Timepoint X Genotype interaction	-0.011	0.012	9688.000	-0.921	0.357	
		linear mixed effects interaction model	all	Injury group (mTBI vs sham)	0.139	0.065	73.940	2.135	0.036	*
				Timepoint (weeks)	-0.004	0.005	9661.000	-0.763	0.446	
				Genotype (OPC-MyrfKO vs OPC-MyrfKO-CTRL)	-0.009	0.065	75.020	-0.143	0.887	
				Sex (M vs F)	-0.127	0.054	34.480	-2.361	0.024	*
				Stimulus duration (s)	0.752	0.060	9794.000	12.478	<2e-16	***
				pre-mTBI incorrect latency	0.017	0.034	33.740	0.482	0.633	
				Injury group X Timepoint interaction	-0.010	0.006	9688.000	-1.672	0.095	.
				Genotype X Timepoint interaction	0.008	0.006	9684.000	1.379	0.168	
		linear mixed effects covariate model	all	Injury group (mTBI vs sham)	0.077	0.053	34.700	1.443	0.158	
				Timepoint (weeks)	-0.005	0.003	9705.000	-1.794	0.073	.
				Genotype (OPC-MyrfKO vs OPC-MyrfKO-CTRL)	0.042	0.053	34.750	0.785	0.438	
				Sex (M vs F)	-0.129	0.054	34.520	-2.393	0.022	*
				Stimulus duration (s)	0.753	0.060	9796.000	12.483	<2e-16	***
				pre-mTBI incorrect latency	0.015	0.034	33.780	0.451	0.655	
S6	Reward collection latency (s)	linear mixed effects 3-way interaction model	all	Injury group (mTBI vs sham)	-0.007	0.066	58.800	-0.104	0.917	
				Timepoint (weeks)	0.003	0.003	18520.000	0.967	0.334	
				Genotype (OPC-MyrfKO vs OPC-MyrfKO-CTRL)	0.153	0.072	60.930	2.124	0.038	*
				Sex (M vs F)	-0.108	0.046	33.170	-2.330	0.026	*
				Stimulus duration (s)	0.025	0.026	18530.000	0.963	0.336	
				pre-mTBI reward collection latency	0.178	0.104	40.920	1.710	0.095	.
				Injury group X Timepoint interaction	0.003	0.004	18520.000	0.712	0.476	
				Injury group X Genotype interaction	-0.014	0.097	58.800	-0.141	0.889	
				Genotype X Timepoint interaction	-0.014	0.005	18520.000	-3.032	0.002	**
				Injury group X Timepoint X Genotype interaction	0.011	0.006	18530.000	1.746	0.081	.
		linear mixed effects interaction model	all	Injury group (mTBI vs sham)	-0.013	0.048	59.250	-0.261	0.795	
				Timepoint (weeks)	0.000	0.003	18520.000	0.119	0.905	
				Genotype (OPC-MyrfKO vs OPC-MyrfKO-CTRL)	0.145	0.048	62.650	3.003	0.004	**
				Sex (M vs F)	-0.105	0.046	33.600	-2.296	0.028	*
				Stimulus duration (s)	0.026	0.026	18530.000	0.975	0.330	
				pre-mTBI reward collection latency	0.180	0.103	41.490	1.743	0.089	.
				Injury group X Timepoint interaction	0.008	0.003	18520.000	2.582	0.010	**

				Genotype X Timepoint interaction	-0.008	0.003	18530.000	-2.590	0.010	**
		linear mixed effects covariate model	1 week	Injury group (mTBI vs sham)	0.048	0.056	43.031	0.871	0.388	
				Genotype (OPC-MyrfKO vs OPC-MyrfKO-CTRL)	0.167	0.055	43.310	3.008	0.004	**
				Sex (M vs F)	-0.046	0.056	42.980	-0.817	0.419	
				Stimulus duration (s)	0.161	0.040	4613.499	4.050	0.000	***
				pre-mTBI reward collection latency	0.355	0.131	45.949	2.717	0.009	**
			4 weeks	Injury group (mTBI vs sham)	-0.042	0.048	38.327	-0.868	0.391	
				Genotype (OPC-MyrfKO vs OPC-MyrfKO-CTRL)	0.088	0.049	39.809	1.794	0.080	.
				Sex (M vs F)	-0.081	0.052	36.782	-1.558	0.128	
				Stimulus duration (s)	-0.090	0.059	4432.728	-1.529	0.126	
				pre-mTBI reward collection latency	0.169	0.117	40.818	1.446	0.156	
			8 weeks	Injury group (mTBI vs sham)	0.045	0.059	42.784	0.765	0.448	
				Genotype (OPC-MyrfKO vs OPC-MyrfKO-CTRL)	0.027	0.059	42.846	0.453	0.653	
				Sex (M vs F)	-0.118	0.060	42.752	-1.964	0.056	.
				Stimulus duration (s)	-0.011	0.026	4652.640	-0.430	0.667	
				pre-mTBI reward collection latency	0.121	0.137	43.417	0.884	0.382	
			12 weeks	Injury group (mTBI vs sham)	0.133	0.076	36.494	1.756	0.088	.
				Genotype (OPC-MyrfKO vs OPC-MyrfKO-CTRL)	0.103	0.076	38.613	1.359	0.182	
				Sex (M vs F)	-0.186	0.079	28.533	-2.362	0.025	*
				Stimulus duration (s)	0.022	0.073	4754.235	0.308	0.758	
				pre-mTBI reward collection latency	0.067	0.177	35.716	0.382	0.705	
S6	Latency (s)	linear mixed effects 3-way interaction model	all	Trial (incorrect vs correct)	0.711	0.020	1309.000	35.303	<2e-16	***
				Injury group (mTBI vs sham)	0.033	0.068	138.800	0.477	0.634	
				Timepoint (weeks)	-0.012	0.005	1263.000	-2.527	0.012	*
				Genotype (OPC-MyrfKO vs OPC-MyrfKO-CTRL)	0.014	0.071	131.000	0.201	0.841	
				Sex (M vs F)	-0.053	0.040	57.460	-1.325	0.190	
				Stimulus duration (s)	0.279	0.038	1258.000	7.237	0.000	***
				Injury group X Timepoint interaction	-0.002	0.007	1262.000	-0.311	0.756	
				Injury group X Genotype interaction	0.062	0.099	133.800	0.622	0.535	
				Genotype X Timepoint interaction	0.006	0.007	1260.000	0.949	0.343	
				Injury group X Timepoint X Genotype interaction	-0.003	0.009	1260.000	-0.341	0.733	
		linear mixed effects interaction model	all	Trial (incorrect vs correct)	0.711	0.020	1311.000	35.316	<2e-16	***
				Injury group (mTBI vs sham)	0.062	0.049	137.300	1.256	0.211	
				Timepoint (weeks)	-0.011	0.004	1263.000	-2.711	0.007	**
				Genotype (OPC-MyrfKO vs OPC-MyrfKO-CTRL)	0.046	0.049	136.500	0.926	0.356	
				Sex (M vs F)	-0.052	0.040	58.040	-1.300	0.199	
				Stimulus duration (s)	0.278	0.038	1259.000	7.239	0.000	***
				Injury group X Timepoint interaction	-0.004	0.005	1261.000	-0.766	0.444	
				Genotype X Timepoint interaction	0.005	0.005	1261.000	1.010	0.313	
		linear mixed effects covariate model	all	Trial (incorrect vs correct)	0.710	0.020	1313.000	35.308	<2e-16	***
				Injury group (mTBI vs sham)	0.039	0.040	58.050	0.995	0.324	
				Genotype (OPC-MyrfKO vs OPC-MyrfKO-CTRL)	0.075	0.040	57.880	1.898	0.063	.
				Timepoint (weeks)	-0.010	0.002	1263.000	-4.452	0.000	***
				Sex (M vs F)	-0.052	0.040	58.040	-1.294	0.201	
				Stimulus duration (s)	0.279	0.038	1261.000	7.241	0.000	***
S7	Ymaze total number of alternations	linear mixed effects interaction model	1, 2, 6, and 12 weeks	Genotype (OPC-MyrfKO vs OPC-MyrfKO-CTRL)	-2.181378	1.560035	139.380252	-1.398	0.1642	
				Timepoint (weeks)	-0.03028	0.012661	217.000001	-2.392	0.0176	*
				Sex (M vs F)	-2.902792	1.247547	69.999997	-2.327	0.0229	*
				Genotype X Timepoint interaction	0.008738	0.020819	217.000001	0.42	0.6751	
		linear mixed effects covariate model	1, 2, 6, and 12 weeks	Genotype (OPC-MyrfKO vs OPC-MyrfKO-CTRL)	-1.8144	1.29196	70	-1.404	0.16463	
				Timepoint (weeks)	-0.02705	0.01003	218	-2.696	0.00756	**
				Sex (M vs F)	-2.90279	1.24755	70	-2.327	0.02288	*
